# Supplementary material for: Content-rich biological network constructed by mining PubMed abstracts
Source: BMC Bioinformatics. 2004 Oct 8;5:147. doi: 10.1186/1471-2105-5-147 (PMC528731; doi:10.1186/1471-2105-5-147)
Supplement: Additional File 5 — The original Chilibot query results of the term "long-term potentiation (LTP)" and 22 other terms, limiting the latest references analyzed to the years 1990, 1995, 2000, and 2004. [file 1471-2105-5-147-S5.bz2 › chilibotAdditionalFile5/ltp2004/html/TRKA_ERK.html]

 


 **TRKA** and **ERK** 
  
Found 55 abstracts in PubMed,  **30 abstracts were retrieved and analyzed**.  


---

 Search Google  |
 PDF files only 
|  EDU domain only 

---

**Interactive relationship** (e.g. stimulation, inhibition, etc)

- AEA thus inhibited  **TrkA**  induced Rap1 B Raf  **ERK**  activation.  Ref: 12237305 J Biol Chem, 2002
- Overall, DQ elicited the differentiation of PC12 cells through Trk A  [ **TRKA** ]  phosphorylation followed by  **Erk**  1 2 and PI3 kinase activation.  Ref: 14706634 Biochem Biophys Res Commun, 2004
- The  **trkA**  and  **ERK**  1 2 activation were corroborated using phospho specific antibodies against  **trkA**  phosphorylated on Tyr 490, and antibodies against Thr Tyr phosphorylated  **ERK**  1 2.  Ref: 11336640 Biochem J, 2001
- However, basic fibroblast growth factor bFGF induced rapid phosphorylation of Erk1 2, and 3H thymidine incorporation in NIH3T3, WT11, single mutant SM, and double mutant DM cells, suggesting that the impaired NGF induced  **Erk**  phosphorylation and thymidine incorporation observed in DM cells are due to the double cysteine mutations in the  **trkA**  receptor.  Ref: 11859925 J Mol Neurosci, 2001
- Cumulatively, our findings support a model in which Cys436 of the  **trkA**  is responsible for the rapid transfer of the transmembrane occupancy signal to the SHC adaptor protein for activation of the Ras  **Erk**  pathway and DNA synthesis.  Ref: 11859925 J Mol Neurosci, 2001
- We show that extracellular signal regulated kinase  **Erk**  acts as an intermediate in protein kinase C regulated  **TrkA**  cleavage.  Ref: 12058067 Mol Biol Cell, 2002

**Parallel relationship** (e.g. studied together, co-existance, homology, etc.)

- Using immunoprecipitation assays against phosphotyrosine residues, time and dose dependent increases in tyrosine phosphorylation were observed in the  **trkA**  receptor, PLCgamma 1 and  **ERK**  1 2.  Ref: 11336640 Biochem J, 2001
- In contrast to the  **Erk**  route, the p38 MAPK was able to stimulate  **TrkA**  cleavage in cells devoid of TACE activity, indicating that other proteases are also involved in  **TrkA**  shedding.  Ref: 12058067 Mol Biol Cell, 2002
- C6 ceramide induces  **TrkA**  phosphorylation and selective activation of the phosphatidyl inositol 3 kinase PI3 kinase Akt pathway but not the MAPK  **ERK**  pathway.  Ref: 14614956 Neuropharmacology, 2003
- Differential activation of the  **TrkA**  phosphorylated mitogen activated protein MAP extracellular signal regulated kinase  **ERK**  kinase 1 phosphorylated  **ERK**  phosphorylated cAMP responsive element binding protein and  **TrkA**  phosphorylated MAP  **ERK**  kinase 3 6 phosphorylated p38 MAP signal transduction pathways, also suppressible by K252alpha, occurs in the two cell types.  Ref: 12208732 Cancer Res, 2002
- In B5P cells, a clonal cell line of PC12 cells overexpressing tyrosine kinase NGF receptor  **TrkA** , cPA elicited transphosphorylation of  **TrkA** . cPA elicited  **ERK**  activation was blocked by K252a and PKI.  Ref: 14622107 J Neurochem, 2003
- At the molecular level, NS 1231 enhanced NGF induced signalling events, such as  **TrkA**  phosphorylation at the Shc binding site Tyr490 as well as  **ERK**  activation in PC12 cells.  Ref: 12067229 J Neurochem, 2002
- A double cysteine  **trkA**  mutant exhibiting reduced NGF binding and delayed  **Erk**  signaling.  Ref: 11859925 J Mol Neurosci, 2001
- Overexpression of bcl 2 also leads to increased expression of  **TrkA**  and increased phosphorylation of signal transductants in, albeit not specific for, the  **TrkA**  MEK  **ERK**  pathway.  Ref: 12934111 Oncogene, 2003
